# The knowledge, attitude and behavior about public health emergencies and the response capacity of primary care medical staffs of Guangdong Province, China

**DOI:** 10.1186/1472-6963-12-338

**Published:** 2012-09-25

**Authors:** Zhou Zhiheng, Wang Caixia, Wang Jiaji, Yang Huajie, Wang Chao, Liang Wannian

**Affiliations:** 1 School of public health and family medicine, Capital medical university of China, No.10 Xitoutiao, You An Men, Beijing, 100069, P.R. China; 2 School of public health, Guangzhou medical university, Guangzhou, China; 3 Department of internal medicine of the first hospital of Guangzhou city, Guangzhou, China

**Keywords:** Primary care medical staffs, Public health emergencies, Knowledge, Attitude and behavior, Emergency response capacity

## Abstract

**Background:**

Primary care medical staffs’ knowledge, attitude and behavior about health emergency and the response capacity are directly related to the control and prevention of public health emergencies. Therefore, it is of great significance for improving primary care to gain in-depth knowledge about knowledge, attitude and behavior and the response capacity of primary care medical staffs. The main objective of this study is to explore knowledge, attitude and behavior, and the response capacity of primary care medical staffs of Guangdong Province, China.

**Methods:**

Stratified clustered sample method was used in the anonymous questionnaire investigation about knowledge, attitude and behavior, and the response capacity of 3410 primary care medical staffs in 15 cities of Guangdong Province, China from July, 2010 to October 2010. The emergency response capacity was evaluated by 33 questions. The highest score of the response capacity was 100 points (full score), score of 70 was a standard.

**Results:**

62.4% primary care medical staffs believed that public health emergencies would happen. Influenza (3.86 ± 0.88), food poisoning (3.35 ± 0.75), and environmental pollution events (3.23 ± 0.80) (the total score was 5) were considered most likely to occur. Among the 7 public health emergency skills, the highest self-assessment score is “public health emergency prevention skills” (2.90 ± 0.68), the lowest is “public health emergency risk management (the total score was 5)” (1.81 ± 0.40). Attitude evaluation showed 66.1% of the medical staffs believed that the community awareness of risk management were ordinary. Evaluation of response capacity of health emergency showed that the score of primary care medical staffs was 67.23 ± 10.61, and the response capacity of senior physicians, public health physicians and physicians with relatively long-term practice were significantly better (P <0.05). Multiple linear stepwise regression analysis showed gender, title, position, type of work, work experience and whether to participate relative training were the main factors affecting the health emergency response capacity.

**Conclusions:**

The knowledge, attitude and behavior about public health emergencies and the response capacity of primary care medical staffs of Guangdong Province (China) were poor. Health administrative departments should strengthen the training of health emergency knowledge and skills of the primary care medical staffs to enhance their health emergency response capabilities.

## Background

In recent years, the world's public health emergencies continue to occur and seriously affect people's health physically and mentally. Among all public health emergency response agencies, hospital is the key place to provide medical and psychological services [1-3], and medical staffs are the main force involved in response to public health emergencies. The knowledge, skills, attitudes and behavior of the medical staffs have a direct impact on regional or national public health emergency management [4]. Studies have shown that 80% of the world public health emergencies occurred in the community [5], in turn, primary health care institutions plays an important role in the emergency management system and they are the first hurdle to effectively reduce devastation from disasters [6,7]. China is implementing the national policy of "community public health service package" which requires primary health services to handle community public health emergencies collaboratively. However, there were few reports concerning on community health emergencies response in China. Moreover, there were no reports that comprehensive analyzed the knowledge, attitude and behavior, and the response capacity of primary care medical staffs of China. Some published reports were only concerning on the portion of knowledge, attitude and behavior, or response capacity. Studies have indicated that the knowledge, attitude and behavior of primary care medical staffs for public health emergency were not satisfactory, the response capacity of primary care medical staffs are relatively low [3,8-12]. Their knowledge and attitude on emergency reflect their level of theoretical knowledge and belief. Their behavior reflects their practice and experience of public health emergencies. The knowledge, attitude and behavior are the parts of the health emergency response capacity, and directly affect the capability of their response capacity. Therefore, improvement of the emergency response capacity of primary care medical staffs is the key element for handling public health emergencies effectively [13]. It is of great significance to explore knowledge, attitude and behavior, and the response capacity of primary care medical staffs, and analyze the main factors. Here, we investigated the knowledge, attitude and behavior, and the emergency response capacity of 3410 medical staffs from primary care hospitals.

## Methods

### Objects

As an economically developed coastal province of southern China, the first known cases of SARS occurred in Guangdong province, 2003. Since then, its response of health emergency is of great concern. 3410 primary care medical staffs from 120 primary hospitals in 15 cities of Guangdong participated in this study, including doctors, nurses and other medical staff.

### Sampling methods

Stratified random cluster sampling method was used and the regions surveyed were divided into three types of regions based on economic conditions, including developed regions, regions of middle economic level, economically underdeveloped regions. Five cities in each type of region were investigated and 8 primary care hospital (the town hospital or community health service center) of each city were chosen, namely, all medical staffs (except for the staff without job title or support staff ) in the 120 hospital were conducted a questionnaire survey.

### Survey contents

"Health emergency questionnaire" was designed. The retest reliability of pre-survey was 0.801, Cronbach alpha coefficient was 0.928, suggesting that the reliability of the questionnaire was very high. The questionnaire was recognized by health emergency experts and investigators, which suggested that the validity of the questionnaire was ok. The questionnaire included: general conditions, knowledge(including Public health emergency regulations and rules, Concept of risk management, Diagnosis/judge/prevention of public health emergencies, reporting for public health emergencies), attitude(including the awareness of risk management , propaganda of health emergencies, response capacity of health emergencies, the weakest part of response capacity and Main objective factors leading to the poor response capacity of the local community) and behavior(included the frequency of emergency response training since work, Sources to gain health emergency associated knowledge, Response to public health emergencies, and the experiences of treating public health emergency cases at the scene), and the response capacity of public health emergencies, the training needs of emergency response, evaluation of emergency response capacity and associated factors. The assignment method was used in the risk awareness survey of 18 common types of public health emergencies. There were 5 alternative answers to each question and each answer represented a score (definitely will not, 1 point; impossible, 2 points; possible, 3 points; will happen, 4 points; definitely will, 5 points). The higher the score was, the stronger the awareness of risk was. There were 4 answers to each self-assessment question of emergency knowledge and skills (completely do not understand, 1 point; understand a little, 2 points; a relatively better grasp, 3 points; fully grasp, 4 points). A higher score represented a higher level. A questionnaire evaluation of emergency response capacity was performed using a total of 33 questions. The highest score was 100 points (full score), while the lowest score was 33 points, score of 70 was a standard.

### Survey methodology

Trained, qualified investigators were separately sent to each primary health care sector. After gathering the medical staffs in the primary care hospital, the investigators introduced the purpose of this survey and notified the precautions about the questionnaire. Then, the medical staffs surveyed filled the anonymous self-administered questionnaires and the investigators collected the questionnaires at the scene.

### Statistical methods

After checking the questionnaire, epiData 3.0 software was used for the data entry procedure. To ensure the accuracy of data entry, double-entry method was adopted. SPSS 13.0 statistical software (Version13.0; SPSS Inc., Chicago, IL, USA) was applied for statistical analysis. The data are presented as mean values ± standard deviation. Differences of the mean values between groups were analyzed with t test or analysis of variance. Counting data are presented with rate or composition ratio and the differences between groups were compared with chi-square test. Health emergency response capacity associated factors were analyzed using backward linear stepwise regression analysis. P <0.05 was considered statistically different.

### Ethical approval

All research carried out was conducted with integrity and in line with generally accepted ethical principles and approved by Research Ethic Committee of Capital Medical University, Guangzhou Medical University and the First Hospital of Guangzhou, China. We did the survey under the agreement of the medical staffs. All the personal information of the medical staffs involved in the survey was not open.

## Results

### General conditions

3500 survey questionnaires were sent and 3410 valid questionnaires were collected with a recycling rates of 97.4%, of which 3356 were male (33.9%), 2254 were female (66.1%). The age was 32.47 ± 8.83 (ranged from 18–72 years of age). The detailed information of the medical staff were as follows, community general practitioners, 934 people (accounting for 27.4%), medical specialists, 1026 (30.1%), community public health physicians, 109 (3.2%), nurses, 791 (23.2%), other health professionals, 550 (16.2%); The composition included junior, 1995 (58.5%), intermediate grade, 771 (22.6%), senior, 644 (5.6%). The education composition was as follows: 689 people of secondary education (20.2%), 1449 people with college degree (42.5%), 1272 people with a bachelor's degree or higher (37.2%). The length of service of the medical staffs surveyed in primary health care units were 8.35 ± 8.10 years (ranged from 1–45 years). There were 1442 people (42.3%) who worked less than 5 years, 1064 people (31.2%) who worked less than 5–10 years, and 904 people (26.5%) worked for more than 10 years.

### Analysis of primary care medical staffs’; risk awareness of public health emergencies

In our study, the risk awareness was analyzed in two aspects, including the overall risk awareness and the awareness of 18 kinds of public health emergencies. 62.4% of respondents believed that public health emergencies would occur in the locality. The overall risk awareness between medical staffs of different titles and different length of service were significantly different (P <0.05). However, there was no significant difference of the overall risk awareness between staff for different types of jobs and from different regions (P > 0.05, Table 1). In the 18 kinds of public health emergencies (total score was 5), the top three of the risk awareness were influenza (3.86 ± 0.876 points), food poisoning (3.35 ± 0.75 points), and environmental pollution events (3.23 ± 0.80 points). The urban primary hospital medical staff had a better risk awareness of avian influenza and AIDS than medical staffs from rural areas. However, regarding to cholera, typhoid, encephalitis, plague and anthrax, risk awareness of rural medical staff was significantly higher than urban medical staffs (P <0.05). There was no significant difference of risk awareness in the remaining 11 kinds of public health emergencies between rural and urban doctors (P > 0.05, Table 2).

**Table 1:** Comparison of the overall risk awareness of different types of primary health care medical staffs

**Staff**	**definitely will not**	**impossible**	**possible**	**will happen**	**definitely will**	**χ**^**2**^	**P value**
Job title ①Junior(%)	22.1	38.8	30.7	6.5	1.9	32.104	P_total_ = 0.000
②intermediate grade(%)	27.7	21.2	22.2	12.2	9.6		P_①-②_ = 0.000 , P_①-③_ = 0.363
③**senior**(%)	27.3	40.7	25.6	4.7	4.7		P_②-③_ = 0.340
**Job type** ①**Nurses** (%)	26.1	37.9	26.2	6.2	1.6	18.331	P_total_ = 0.106
②**Medical specialists**(%)	25.6	40.8	26.2	5.9	1.4		P_①-②_ = 0.784, P_①-③_ = 0.285
③Community general practitioners(%)	22.4	38.7	32.1	5.2	1.6		P_①-④_ = 0.081, P_②-③_ = 0.784
④Public health physician(%)	19.2	33.7	41.3	3.8	1.9		P_②-④_ = 0.784, P_③-④_ = 0.053
**Length of service** ①**<5 years(%)**	20.6	41.3	30.5	5.9	1.7	43.304	P_total_ = 0.000
②**5-10 years(%)**	32.0	40.0	29.2	6.8	1.1		P_①-②_ = 0.377, P_①-③_ = 0.000
③**>10 years(%)**	30.2	35.2	30.1	2.6	2.0		P_②-③_ = 0.000
**The sources of physicians of primary care hospitals**							
①**Urban Physicians (%)**	24.4	39.0	28.6	6.0	2.0	7.091	P_total_ = 0.131
②**Rural Physicians (%)**	23.1	38.7	31.9	5.0	1.3		
**Summary**	1.6	5.4	30.5	38.8	23.6		

**Table 2:** Comparison of the risk awareness of 18 different types of public health emergencies between urban and rural primary health care medical staffs (χ ± s, the total score is 5)

**Items**	**Urban medical staffs**	**Rural medical staffs**	**Total**	**t**	**P value**
Chemical poisoning	2.86 ± 0.01	2.90 ± 0.02	2.88 ± 0.61	1.884	0.060
Food poisoning	3.36 ± 0.73	3.33 ± 0.77	3.35 ± 0.75	−1.131	0.258
Radiological accidents	2.54 ± 0.70	2.58 ± 0.70	2.56 ± 0.70	1.548	0.122
Environmental pollution	3.21 ± 0.76	3.25 ± 0.85	3.23 ± 0.78	1.309	0.191
Nuclear accident	2.05 ± 0.81	2.10 ± 0.86	2.07 ± 0.83	1.833	0.067
Zoonosis	3.01 ± 0.88	3.03 ± 0.63	3.02 ± 0.65	1.130	0.259
Influenza	3.87 ± 0.88	3.85 ± 0.88	3.86 ± 0.88	−0.384	0.701
Cholera	2.84 ± 0.68	2.89 ± 0.65	2.87 ± 0.67	2.182	0.029
Typhoid	2.93 ± 0.64	2.98 ± 0.62	2.95 ± 0.63	2.328	0.020
Avian Influenza	3.07 ± 0.54	3.03 ± 0.57	3.05 ± 0.56	−2.031	0.042
Epidemic encephalitis	2.98 ± 0.56	3.01 ± 0.57	2.99 ± 0.57	1.146	0.252
Epidemic encephalitis B	2.97 ± 0.55	3.04 ± 0.62	3.00 ± 0.58	3.914	0.000
SARS	2.88 ± 0.56	2.86 ± 0.57	2.87 ± 0.57	−1.027	0.304
Plague	2.62 ± 0.67	2.68 ± 0.67	2.65 ± 0.67	2.310	0.021
Anthrax	2.51 ± 0.68	2.58 ± 0.67	2.54 ± 0.67	2.999	0.003
AIDS	3.22 ± 0.75	3.13 ± 0.78	3.15 ± 0.75	−3.651	0.000
Unknown infectious diseases	3.09 ± 0.55	3.08 ± 0.56	3.09 ± 0.56	−0.742	0.458
Other infectious diseases	3.14 ± 0.57	3.16 ± 0.57	3.13 ± 0.57	−0.315	0.753

### Medical staffs self-assessment of emergency knowledge and skills

Self-assessment was performed to investigate the health emergency knowledge and skills of primary care medical staffs. Regarding to the grasp of emergency knowledge, most primary care medical staffs (52.5%) mastered the public health department to report, while the number of medical staffs grasping the concept of risk management were the least (11.0%). In seven emergency response skills(total score was 5), the highest score based on the self-assessment questionnaires was the public health emergency prevention skills (2.90 ± 0.67) and the lowest score was the public health emergency risk management (1.81 ± 0.40). Based on the self-assessment results, the skills of urban medical staffs, including the grasp of 4 kinds of emergency-related knowledge, first aid of public health emergency, public health emergency prevention, monitoring of public health emergencies, plan making for public health emergency, communication during a public health emergency were significantly higher than rural medical staffs (P <0.05). The grasp of epidemiological investigation and public health emergency risk management showed no statistically difference between urban and rural doctors (P > 0.05, Table 3).

**Table 3:** Comparison of self-assessment of emergency health knowledge and skills between urban and rural primary care medical staffs

**Items**	**Urban medical staffs**	**Rural medical staffs**	**Total**	**χ**^**2**^**/t**	**P value**
**Fully grasp (%)**					
Public health emergency regulations	22.1	16.2	19.5	23.657	0.000
Concept of risk management	11.6	10.3	11.0	7.470	0.024
Diagnosis/judge of public health emergencies	24.2	16.6	21.0	29.117	0.000
Public health department to report	57.0	46.5	52.5	36.850	0.000
Time limit of reporting	52.6	42.4	48.2	34.881	0.000
**Scores of skills (points, a total of 5)**					
First aid of public health emergency	2.97 ± 0.68	2.91 ± 0.68	2.94 ± 0.68	−2.632	0.009
Public health emergency prevention	2.93 ± 0.67	2.87 ± 0.68	2.90 ± 0.67	−2.330	0.020
Epidemiological investigation	2.72 ± 0.72	2.75 ± 0.68	2.73 ± 0.70	1.360	0.174
Risk management of public health emergencies	1.80 ± 0.40	1.81 ± 0.39	1.81 ± 0.39	0.510	0.610
Monitoring of public health emergencies	2.57 ± 0.81	2.51 ± 0.78	2.55 ± 0.80	−2.335	0.020
Plan making for public health emergenc	2.59 ± 0.81	2.53 ± 0.81	2.56 ± 0.81	−2.154	0.031
Communication during public health emergencies	2.60 ± 0.81	2.55 ± 0.80	2.58 ± 0.81	−1.871	0.061

### Attitude of primary care medical staffs towards issues related to public health emergencies

The attitude evaluation showed 66.1% of the medical staffs believed that the community awareness of risk management were ordinary. 71.1% of the medical staffs considered that the propaganda of health emergency was not doing enough. 71.9% of the medical staffs suggested that the response capacity of health emergency was normal. 46.7% of the medical staffs believed that it would show the weakest response capacity when facing diseases of unknown causes. About 80% of people considered that the main objective reasons that led to the poor response capacity of primary hospital emergency were a shortage of staff (83.0%),a lack of equipment (80.6%) and aging equipment (77.6%). The vast majority of medical staffs thought that the primary hospital should be involved in early warning and monitoring, assisting epidemiological investigations and providing emergency training (Table 4).

**Table 4:** The attitude of primary care medical staffs towards public health emergencies associated problems

**Items**	**Urban medical staffs**	**Rural medical staffs**	**Total**	**χ**^**2**^	**P value**
**Awareness of risk management**					
Strong(%)	23.9	23.6	23.8	1.657	0.437
Normal(%)	66.5	65.4	66.1	0.435	0.510
Weak(%)	9.6	10.9	10.1	1.513	0.219
**Propaganda of health emergencies**					
Poor(%)	4.1	3.4	4.0	1.048	0.306
Not enough(%)	69.5	73.3	71.1	5.770	0.016
Enough (%)	26.1	23.3	24.9	3.627	0.057
**Response capacity of health emergencies**					
Strong(%)	17.2	16.5	16.9	0.392	0.822
Normal(%)	71.8	71.9	71.9	0.007	0.931
Weak(%)	11.0	11.5	11.2	0.213	0.644
**The weakest part of response capacity**					
Response to important infectious diseases(%)	28.4	29.2	28.7	0.271	0.603
Response to diseases of unknown cause(%)	46.2	47.4	46.7	0.463	0.496
Response to major food poisoning(%)	6.1	7.8	6.8	3.942	0.047
0.021Response to occupational poisoning(%)	15.6	12.8	14.4	5.309	0.021
Others(%)	3.8	2.8	3.4	2.728	0.099
**Main objective factors leading to the poor response capacity (multiple choices)**					
a shortage of staff(%)	83.9	78.6	83.0	6.799	0.147
a lack of equipment(%)	80.9	80.3	80.6	8.228	0.084
aging equipment(%)	77.7	77.5	77.6	2.318	0.678
Primary hospital should be involved in warning and monitoring (%)	84.9	88.8	86.6	34.359	0.000
should assist epidemiological investigations(%)	90.5	88.5	89.7	11.228	0.024
should provide emergency training(%)	87.1	83.0	85.3	13.131	0.011

### Behavior of primary care medical staffs towards public health emergencies

Regarding to behavior, 33.3% of the medical staffs has never participated in the training of health and the rate of rural medical staffs was significantly higher than urban doctors (P < 0.05). In addition, organizational learning unit (38.4%), media (32.4%) and the accumulation of practical work (31.6%) were their main sources to gain health emergency associated knowledge. 25.7% of respondents had participated in treatment of public health emergency cases at the scene, and it showed no significant difference between urban and rural medical staffs (P>0.05). 57.3% of the medical staffs believed that they would carry out mutual aid work when facing public health emergencies. The rate of "mutual aid" in rural medical staffs was significantly higher than in urban medical staffs (P <0.05). In the mean time, 39.4% of the medical staffs suggested that they would carry out self-aid and the percentage of urban medical staffs choosing "self-aid" was significantly higher than that of rural medical staffs (P <0.05, Table 5).

**Table 5:** Behavior analysis of primary care medical staffs towards public health emergencies

**Items**	**Urban medical staffs**	**Rural medical staffs**	**Total**	**χ**^**2**^	**P value**
**The frequency of emergency response training since work**					
Once a year(%)	32.5	29.7	31.3	3.014	0.083
Once every 2 to 5 years(%)	30.0	20.3	25.8	40.982	0.000
Interval of more than 5 years(%)	9.3	10.0	9.6	0.453	0.501
Never (%)	28.2	40.1	33.3	53.279	0.000
**Sources to gain health emergency associated knowledge(multiple choice)**					
School education(%)	25.2	29.2	26.9	8.695	0.003
Self-learning(%)	17.7	18.7	18.1	0.570	0.450
Organizational learning unit(%)	40.3	35.8	38.4	7.013	0.008
Media(%)	38.6	27.3	32.4	47.763	0.000
Continuing education(%)	25.1	15.6	20.5	45.927	0.000
Accumulation of practical work(%)	29.2	36.1	31.6	18.290	0.000
Other sources(%)	17.2	23.0	19.3	16.831	0.000
Participated in treatment at the scene(%)	25.2	26.3	25.7	0.510	0.475
**Response to public health emergencies(Single-choice)**					
Self-aid(%)	45.9	31.0	39.4	83.743	0.000
Mutual-aid(%)	51.7	64.5	57.3	56.314	0.000
Escape(%)	1.5	1.9	1.7	0.847	0.358
Don’t know what to do(%)	0.9	2.5	1.6	14.410	0.000

### Training needs analysis of primary care medical staffs towards public health emergencies

Training needs analysis indicated that 78.4% of the medical staffs believed it was necessary to implement public health emergency training for all medical staff. 8.8% of the respondents indicated that the infectious diseases medical staffs should be trained (Ranked second according to the analysis). The most needed training contents were emergency health laws and regulations (54.8%), risk management (50.0%) and health emergency monitoring and early warning (45.5%).Rural primary care medical staffs had significant higher training needs of risk management, monitoring and early warning, plan making, media communication than urban doctors. However, there was significant difference of training needs of the epidemiological investigation between rural and urban medical staffs and it showed higher needs in urban practitioners (P <0.05). Health care medical staffs suggested that the top three appropriate training methods were practical exercise (27.3%), academic lectures (21.4%) and desktop deduction (19.7%), as shown in Table 6.

**Table 6:** Training needs analysis of primary care medical staff towards public health emergencies

**Items**	**Urban medical staffs**	**Rural medical staffs**	**Total**	**χ**^**2**^	**P value**
**The most needed physicians that should be trained**					
All medical staff(%)	78.2	78.6	78.4	0.079	0.778
Infectious diseases physicians(%)	8.5	9.1	8.8	0.376	0.540
Occupational diseases physicians(%)	1.9	3.1	2.4	4.721	0.030
Emergency Doctors(%)	5.2	3.0	4.2	8.308	0.004
Personnel in Disease Control Agency(%)	5.6	6.1	5.8	0.376	0.540
Other staff(%)	0.6	0.1	0.4	4.763	0.029
**Necessary training contents (Multiple choices)**					
Emergency health laws and regulations(%)	48.3	52.1	50.0	4.124	0.534
General First-Aid Principles(%)	35.0	28.7	32.4	10.791	0.214
Knowledge of risk self-protection(%)	47.3	35.2	42.1	10.013	0.188
Epidemiological investigation(%)	35.4	25.5	31.1	13.428	0.037
Risk management(%)	51.8	58.7	54.8	101.623	0.000
Monitoring and warning(%)	42.2	49.8	45.5	91.596	0.000
Plan making(%)	29.6	38.4	33.4	65.091	0.000
Media communication(%)	20.6	29.2	24.3	45.522	0.000
**The most appropriate training methods (Single choice)**					
Full-time study(%)	10.9	11.2	11.1	29.082	0.000
Academic lectures(%)	21.1	21.9	21.4	0.315	0.575
Case Study(%)	15.8	20.9	18.0	0.096	0.757
Practical exercise(%)	29.8	24.0	27.3	12.235	0.000
Desktop deduction(%)	20.5	18.7	19.7	1.401	0.237
Distance Education(%)	1.7	2.7	2.1	4.132	0.042
Other methods(%)	0.3	0.5	0.4	0.271	0.603

### Analysis of health emergency response capacity based on the questionnaire results

Both assessment questionnaire scoring and self-assessment scoring of the overall emergency response capacity were used for the evaluation of the emergency response capacity of medical staffs. Evaluation of response capacity of health emergencies (A total of 100 points) showed that the score of primary care medical staffs was 67.23 ± 10.61, and the response capacity was related to their titles and working experience or length of service. The scores of response capacity of different job types ranked as follows, public health physicians > community general practitioners > medical specialists > nurses > other medical staff and the scores between different groups were statistically significant (P <0.05). However, there was no significant difference between rural and urban medical staffs (P >0.05, Table 7) Self-assessment of health emergency response capacity indicated that 15.2% of the medical staffs believed that they had a strong response capability. 70.8% of the respondents felt their response capacity was general and there were 14.0% of the medical staffs having poor health emergency response capacity. The results of assessment questionnaire scoring and self-assessment of the overall emergency response capacity were positively correlated (r = 0.48, P = 0.000).

**Table 7:** Assessment of health emergency capacity of different types of physicians

**Physicians**	**Scores (χ ± s, full score = 100)**	**F /t value**	**P value**
**Titles**			
Junior	66.61 ± 10.73	51.337	**P**_**total**_ = 0.000
Intermediate grade	69.40 ± 9.74		**P**_①-②_ = 0.000,**P**_①-③_ = 0.000
Senior	72.92 ± 10.30		**P**_②-③_ = 0.000
**Job type**			
Other medical staff	63.86 ± 12.10	24.236	**P**_**total**_ = 0.000
Nurse	67.560 ± 9.93		**P**_①-②_ = 0.000,**P**_①-③_ = 0.000,**P**_①-④_ = 0.000
Medical specialists	66.80 ± 10.30		**P**_①-⑤_ = 0.000,**P**_②-③_ = 0.143,**P**_②-④_ = 0.022
Community general practitioners	68.77 ± 9.92		**P**_②-⑤_ = 0.000,**P**_③-④_ = 0.000,**P**_③-⑤_ = 0.000
Public health physicians	72.55 ± 11.03		**P**_④-⑤_ = 0.001
**Length of service**			
<5 years	65.57 ± 10.80	35.592	**P**_**total**_ = 0.000
5-10 years	68.41 ± 9.91		**P**_①-②_ = 0.000,**P**_①-③_ = 0.000
>10 years	69.24 ± 10.46		**P**_②-③_ = 0.009
**The sources of the physicians**			
Urban	67.48 ± 10.56	1.649	0.099
Rural	66.85 ± 10.80		
**Summary**	67.23 ± 10.61		

### Analysis of health emergency response capacity associated factors

Stepwise multiple regression analysis was performed. The dependent variable (y) represented the score of response capacity. Independent variables were age(x _1_), gender (x _2_), education(x _3_),title(x _4_), position (x _5_), job type (x _6_),length of service(x _7_), sources(x _8_),emergency training (x _9_). The regression equation was written as y = 59.712 + 7.331 x_9_ + 3.244 x_5_ + 1.881 x_6_-1.080 x_2_ + 1.045 x_4_ + 0.733 x_7_, as shown in Table 8.

**Table 8:** Stepwise multiple regression analysis of health emergency response capacity

**Items**	**B**	**S.E**	**Beta**	**t value**	**P value**
Constant	59.712	1.186	**0.368**	50.357	0.000
Gender	−1.080	0.443	−0.049	−2.439	0.015
Title	1.045	0.370	0.061	2.825	0.005
Position	3.244	0.580	−0.109	5.549	0.000
Job type	1.881	0.396	0.095	4.750	0.000
Length of service	0.733	0.252	0.057	2.915	0.004
Emergency training	7.331	0.415	0.336	17.676	0.000

Note: The assignment was as follows: Sex: 1 male, 2 female; Title: 1 junior, 2 intermediate grade, 3 senior; Position: 0 ordinary staff;1 the head of the hospital or department; job type: 1 primary care medical staffs who were not responsible for community public health work, 2 primary care medical staffs who were responsible in part for community public health work; 3 primary care medical staffs who were responsible for community public health work; length of service: 1, less than 5 years; 2, 5–10 years; 3, more than 10 years; emergency training: 0 did not attend.1 participated.

## Discussion

Primary care medical staffs are gatekeepers to health care success and are also the first response to public health emergency. They play an important role in public health emergency preparedness and response [13]. Besides, their knowledge, attitude and behavior about health emergencies and the response capacity are directly related to the control and prevention of public health emergencies. However, research have indicated that primary care medical staffs of many countries are not ready to deal with the public health emergencies [8,9,14,15], which mainly due to the lack of training and experience about public health emergencies [16-18]. The public health emergencies are usually sudden, unpredictable and with considerable severity. Thus, it put forward higher requirements to the primary care professionals and health institutions to prevent and control the emergencies [19]. There were only a few reports about health emergencies response in China. In our study, anonymous questionnaire investigation of 3410 primary care medical staffs of Guangdong was performed to explore their risk awareness, knowledge, attitude and behavior, training needs about emergency response, the emergency response capacity and its main related factors.

Of the 3410 doctors surveyed, primary care medical staffs were mostly women and represented a shortage of medical staffs with higher titles or education. Regarding to risk awareness, 62.4% of the medical staffs believed that public health emergencies would happen in the region. Influenza (3.86 ± 0.88), food poisoning (3.35 ± 0.75), and environmental pollution events (3.23 ± 0.80) were considered most likely to occur in 18 kinds of public health emergencies (the total score was 5). These results suggested the low awareness of risk of medical staffs in Guangdong. The urban primary hospital medical staff had a poor risk awareness of cholera, typhoid, encephalitis, plague and anthrax and the rural medical staffs were lack of risk awareness about avian influenza and AIDs, which was similar to the results of Jiangou Shen [20]. Risk awareness is the starting point of crisis early warning. A low awareness of risk would affect collection of information related to the crisis, in turn, hampers the identification and early warning [21]. Therefore, the cultivation of a sense of crisis should be strengthened.

Furthermore, the investigation results of knowledge, attitude and behavior of primary care medical staffs were not optimistic. Regarding to the grasp of emergency knowledge, only 11.0% of the respondents knew the concept of risk management and 24.2% of the medical staffs grasped the diagnosis/judge of public emergencies. The total score of emergency response skills was 4 and all the skills surveyed were less than 3. The skills of public health emergency risk management, monitoring and warning, plan making were the weakest. Only a few medical staffs had the response skills of public health emergencies. Most medical staffs grasped only a small part of the knowledge and skills related to emergency response. The attitude evaluation showed 66.1% of the medical staffs believed that the community awareness of risk management were ordinary. 71.1% of the medical staffs considered that the propaganda of health emergency was not doing enough. 71.9% of the medical staffs suggested that the response capacity of health emergency was ordinary and it would show the weakest response capacity when facing diseases of unknown causes. Only 25.7% of respondents had participated in treatment of public health emergency cases which indicated the poor response capacity and the lack of experience [22,23].

Training plays an important role for the development of public health emergency response capacity [24], but the investigation has shown that the training was apparently not enough. About a third of the respondents have never participated in primary care training and the rate of rural medical staffs (40.1%) was even higher. Training needs analysis indicated that 85.3% of the respondents suggested it was necessary to provide public health emergency training. 78.4% of the medical staffs believed it was necessary to implement training for all medical staff. Health care medical staffs suggested that most appropriate training methods were practical exercise. These indicated a wide demand for training, however the opportunities of training were few and present training is usually lack of new ideas. The physicians are willing to participate in practical training rather than classroom teaching which in accordance with previous reports [25,26].

It has been shown that the response capacity of medical staffs in Beijing [22] was weak which was similar to the results of Guangdong. Evaluation of response capacity of health emergency (the total score of 100 points) showed that the score of primary care medical staff was 67.23 and the response capacity of senior medical staffs, public health physicians and physicians with relatively long-term practice were relatively better. Stepwise multiple regression analysis showed that the influence of the factors related to response capacity was ranked in descending order as follows: emergency response training, length of service, position, job type, gender, title, suggesting that training was the most needed method for improvement of response capacity [24]. In the past, it was mainly required for clinical medical diagnosis and treatment of clinical disease in China, and the requirements of health emergency response capacity were low. Therefore, it is essential to carry out targeted training to enhance the prevention and control capabilities of primary care practitioners. The second main factor was the length, indicating that health emergency response capacity is positively related to the experience of physicians [20]. Position and job type ranked the 3rd and 4th, indicating that the position of a medical staff and his responsibilities directly affected the emergency response capacity. Therefore, the head of the hospital are usually having higher risk awareness. Similarly, public health physicians who are responsible for community public health work show significantly higher response capacity than other health care staff.

Although this study included a large sample and have obtained quantitative results, limitations still exist: the paper only focused on Guangdong Province, China and the results can only applied in China; The results from the questionnaire survey showed the knowledge, attitude, behavior and they may not be consistent with the prevention and control during public health emergencies which need further verification.

## Conclusions

Here, we identified the knowledge, attitude and behavior about public health emergencies and the response capacity of primary care medical staffs of Guangdong Province (China) were in a poor situation. Health administrative departments should strengthen the training of primary care health emergency knowledge and skills, establish a mechanism of regular training and provide them with free health emergency training opportunities to enhance their risk awareness and health emergency response capabilities. There is a perceived need to build an excellent team of primary health care. However, the survey only limited in one province in China, and could partially reflect the situation in China and some of the results need to be further test and by other forms of research.

## Competing interests

We declare no competing interests.

## Authors’ contributions

ZZ participated in the design of the study, and carried out epidemiological investigation and drafted the manuscript. CW, YH, CW participated in epidemiological investigation. WL participated in the design and performed the statistical analysis. JW conceived of the study, and participated in its design and coordination and helped to draft the manuscript. All authors have read and approved the final manuscript.

## Pre-publication history

The pre-publication history for this paper can be accessed here:

http://www.biomedcentral.com/1472-6963/12/338/prepub
